# The importance of comprehensive support based on the three pillars of exercise, nutrition, and sleep for improving core symptoms of autism spectrum disorders

**DOI:** 10.3389/fpsyt.2023.1119142

**Published:** 2023-05-16

**Authors:** Nozomu Yano, Kenji Hosokawa

**Affiliations:** ^1^Graduate School of Health Sciences, Doctoral Course, Kagoshima University Graduate School, Kagoshima, Japan; ^2^Department of Child Care and Education, Odawara Junior College, Nagoya, Japan

**Keywords:** autism, neurodevelopmental disorder, exercise, nutrition, sleep

## Abstract

Autism spectrum disorder (ASD) is classified as a neurodevelopmental disorder. The Diagnostic and Statistical Manual of Mental Disorders (DSM)-V, which first described ASD, lists persistent deficits in social communication and interrelationships, as well as limited and recurrent modes of behavior, interests, and activities as diagnostic items. Until recently, understanding the pathophysiology of ASD has been mostly from a neurophysiological perspective, and interventions have been mostly behavioral and psychological. In recent years, however, it has become clear that ASD also affects many bodily systems, including the immune system, the sensorimotor system, and the gut-brain axis, and that these factors simultaneously influence it. In light of this background, a new “connectivome theory” has been proposed as a hypothesis for understanding ASD. “Exercise,” “nutrition,” and “sleep,” which are discussed in this mini-review, have a particularly strong relationship with the immune, musculoskeletal, and gut systems among the pathologies mentioned in the “connectivome theory,” furthermore, many reports suggest improvements in stereo-responsive behavior and social and communication skills, which are the core symptoms of ASD. In addition, these interventions are characterized by being less subject to location and cost limitations and excel in the continuity of therapeutic intervention, and the three interventions may have a reciprocal positive impact and may function as three pillars to support ASD.

## Introduction

Autism spectrum disorder (ASD) is classified as a neurodevelopmental disorder (NDD) in the Diagnostic and Statistical Manual of Mental Disorders (DSM)-V and International Statistical Classification of Diseases and Related Health Problems (ICD)-11. The DSM-V, which first described ASD, lists persistent deficits in social interaction and communication (Social communication: SC), as well as limited and repetitive patterns of behavior, interests, and activities (Stereotypic behavior: SB) as diagnostic items.

Many hypotheses have been proposed to interpret ASD based on the research findings of each era. For example, the “theory of mind disorder hypothesis” (1), the “synaptic abnormality hypothesis,” which seeks causes in abnormal synaptic connections such as Neuroligin (NLGN) and Neurexin (NRXN), which have been discovered as synaptic adhesion molecules (2), and the “serotonin hypothesis,” which theorizes the pathogenic mechanism of ASDs based on abnormalities in serotonin metabolism found in the brains of ASDs (3).

Although most studies have focused on the neurofunctional aspect of ASDs it has recently become clear that ASDs affect many bodily systems, including the immune system, the sensorimotor system, and the gut-brain axis, and that ASDs are affected by these factors simultaneously. In light of this background, Zoccante et al. (4) proposed a new “connectivome theory” as a hypothesis for understanding ASD.

This mini-review focuses on the topics of “exercise,” “nutrition,” and “sleep” as interventions for the conditions mentioned in the “connectivome theory” that are mainly related to the immune, musculoskeletal, and gut systems and are not limited by location or cost. Exercise, nutrition, and sleep are the three pillars for improving core symptoms of ASD (SB, SC), and the importance of each pillar and their interactions will be briefly introduced.

### Exercise

Autism spectrum disorder also has a high 60–80% rate of motor impairment (5, 6). This is a significant social participation barrier and affects SC and SB (7). Exercise interventions are currently attracting attention due to their cost-effectiveness and multiplicity of effects. Here, we discuss previous studies of exercise interventions targeting SC and SB and present the mechanisms of the efficacy of exercise interventions and recommended parameters for such interventions.

#### Effects of exercise interventions

Various exercise interventions have been conducted to date, including aerobic exercises such as jogging and bicycling, martial arts such as karate and judo, ball-handling exercises such as basketball, swimming, and other physical activities such as dance, yoga, and games. These exercise interventions have commonly shown positive effects on physical and cognitive aspects as well as psychosocial aspects, such as improvements in SC and SB (8, 9).

For example, sports such as badminton are more effective for motivation and increasing exercise persistence than simple exercises such as treadmill running (10). In addition, team sports such as mini-basketball and compound exercises that combine several different exercises have been reported to be highly effective in improving psychosocial functioning because they stimulate more brain regions (11).

Furthermore, the effectiveness of interventions combining exercise with other therapies is now being demonstrated (12). Exercise, in particular, is a fundamental component of health and development, along with nutrition and sleep, and comprehensive support of these components is considered essential in the underlying treatment of ASD symptoms.

#### Mechanisms of improvement with exercise intervention

Various hypotheses have been proposed regarding the mechanisms by which exercise improves core symptoms of ASD, but one primary mechanism is the regulation of metabolism. This involves the transport of trophic factors and neurotransmitters (13–15) and may contribute to improving core symptoms of ASD by temporarily normalizing brain activity.

In addition, metabolic modulation may progressively lead to structural and functional changes in the cranial nervous system. In this regard, a Chinese research team has used brain imaging techniques to find changes in white matter fiber connectivity with exercise intervention (16) and changes in functional brain connectivity such as the default mode network (DNM) and executive control network (ECN) during resting state (17, 18).

Furthermore, Wang et al. (19) reported improved executive function and SC after an exercise intervention similar to that of Yang et al. (18). In addition, a meta-analysis of these previous studies organized the neurological effects of exercise intervention into three categories: (1) stabilization of cortical arousal, (2) normalization of resting-state social brain connections, and (3) efficiency of executive processes (20).

#### Time, frequency, and duration of recommended interventions

The time, frequency, and duration of intervention are essential variables that determine the magnitude and persistence of intervention effects (21). A meta-analysis of the effects of exercise interventions in RCTs reported that the range of time, frequency, and duration of interventions was 30–90 min *2–13 times per week *4–24 week. They further stated that long-term interventions of 45–75 min *1–2 times per week *12 weeks or longer were necessary to increase motor skills (20). similarly stated that long-term interventions are more effective than short-term interventions, recommending interventions of at least 50 min *1–2 times per week *10 weeks or more to produce neurological changes and improved psychosocial functioning.

Nekar et al. (22) stated that “the major limitation of cognitive and social training in children with autism remains the engagement and motivation of the children to participate in the intervention program,” and it is essential to optimize exercise events, content, and teaching methods to increase exercise persistence.

#### Recommended exercise intensity

Exercise intensity is essential for the operation of metabolic and associated neurological mechanisms. Exercise intensity is usually measured by heart rate, and Lang et al. (9) suggest that more intense exercise produces more pronounced effects than gentle exercise. The World Health Organization (WHO) recommends 60–69% of maximal heart rate (MHR = 220 – participant’s age) as moderate to vigorous physical activity (MVPA) (23). In contrast, Tse et al. (24) stated that for children with ASD, more than 50% of MHR should be considered MVPA given their low physical activity levels.

Considering that longer-term interventions can have more significant effects, continuity of exercise should be a priority, and it would be advisable to approach MVPA in stages, with the assessment of rating of perceived exertion (RPE), as physical fitness levels vary from individual to individual.

#### Recommended methods of teaching movement

In addition to core symptoms, children with ASD have low motor function and difficulty with confidence and competence. In light of this, most researchers conducting exercise interventions mention step-by-step instructional methods (small-step instructional methods) and errorless learning based on behavioral findings (11, 19, 22). This is important in avoiding psychological anxiety, frustration, and helplessness in children and enhancing exercise continuity.

Finally, while physical activity has been restricted in recent years by the new coronavirus, online meeting tools have spread rapidly. Taking advantage of this, several researchers have proposed telehealth physical activity support (25–28). This method, in which professional support personnel provides exercise coaching and feedback on results, could be an effective means of increasing home-based exercise practices during the coronavirus.

### Nutrition

Autism spectrum disorders are frequently associated with eating disorders (29, 30). They are characterized by a preference for foods high in calories and low in nutrients and an aversion to fruits, vegetables, and grains (31–34). Due to this, they are deficient in micronutrients such as iron, calcium, iodine, magnesium, selenium, vitamins D, E, B 12, folic acid, and biotin, and the need for nutritional management is undisputed. In addition, adequate intake of nutrients requires the presence of a healthy digestive system for uptake by the body (35).

Here we present interventions for micronutrient deficiencies that may contribute to reducing SB and improving SC, as well as interventions from a brain-gut-microbiota axis perspective.

#### Interventions for micronutrient deficiencies

Vitamin supplementation has been suggested by many studies to be effective in improving metabolic and nutritional status in ASD, including mitigation of glutathione, methylation, sulfation, oxidative stress, NADH, ATP, and NADPH (36). Antioxidant vitamin C supplementation improves SB and decreases autism severity (37), and Vitamin D supplementation, which helps functional protein function, improves autism severity, SC, and SB (38). Some have reported improved SC and SB with a combined supplementation of vitamin B6, which acts as a coenzyme, and magnesium (39). Among these, folic acid has attracted attention due to its important function in metabolism and its importance in ASD. The effects of folic acid supplementation on ASD have been reported to improve SC, SB, and verbal communication disorders (40, 41).

#### Interventions from the brain-gut-microbiota axis perspective

There is bidirectional communication between the gut-brain axis, which can regulate gastrointestinal tract and central nervous system functions, and the gut microbiota is believed to play an important role in regulating this bidirectional signaling (42).

The ketogenic diet (KD) involves a high-fat, adequate-protein, low-carbohydrate diet (43–45). KD may improve core symptoms of ASD by normalizing GABA, improving mitochondrial function, improving inflammatory activity and oxidative stress in the brain, inhibiting the mTOR signaling pathway, and modulating the gut microbiota (46).

A gluten- and casein-free diet (GFCF) removes casein from milk and dairy products and gluten from wheat (47). In a meta-analysis reported by Keller et al. (48), providing the GFCF diet to children and adolescents with ASD is no benefit for clinician-reported core symptoms of autism or parent-reported functioning levels and behavioral difficulties. On the contrary, the GFCF diet may cause adverse gastrointestinal effects (48). On the other hand, a meta-analysis reported in 2022 by Quan et al. (49) showed that the GFCF diet could reduce SB, improve perceptions of ASDs, and is safe (49). More extensive studies are needed to conclude the effectiveness and safety of the GFCF diet.

In the intervention comparing KD and GFCF diets, both KD and GFCF diets showed significant improvement in ASD severity compared to the control group. On the other hand, KD showed better results in cognition and SC compared to the GFCF diet, and the GFCF diet showed better results in SB improvement than KD (both *p* > 0.05) (50).

Gastrointestinal symptoms with ASDs are associated with behavioral disorders, sleep disturbances, and attention problems (51, 52).

Autism spectrum disorders have been shown to have large amounts of harmful bacteria and fewer beneficial bacteria in their intestines compared to typically developing children (53).

As defined by the International Scientific Association for Probiotics and Prebiotics (ISAPP), probiotics are “live microorganisms which when administered in adequate amounts confer a health benefit on the host” New interventions are expected to include prob. and their growth factors, prebiotics (54).

These effects have been reported to include decreased ASD severity, increased attention level, decreased SB, improved SC, and improved gastrointestinal disturbances (55–58).

On the other hand, the results of the meta-analysis showed no significant improvement in ASD severity, gastrointestinal problems, or psychopathology comorbid with ASD. However, they cite the small number of background studies and methodological uncertainty as concerns and conclude that further research on standardized intervention programs is needed (59). And a research protocol for a large-scale randomized controlled trial of probiotics and prebiotics is currently being drafted (60).

We hope that the study will proceed successfully and that it will yield better results. Although we have presented the effectiveness of single nutritional interventions, nutritional interventions need to be comprehensive, observing the individual’s condition.

In a comprehensive nutrition intervention by Adamas et al. that sequentially deployed vitamin and mineral supplementation, essential fatty acid supplementation, Epsom salt baths, carnitine supplementation, digestive enzyme supplementation, GFCF, and healthy eating, the result was a significant improvement in ASD severity, communication, social skills, sensory deficits, and developmental age. It should be noted, however, that in some cases, there were detrimental effects in the process (61).

As we can learn from this excellent study, we will need to pay attention to the eating disorders of ASDs and comprehensive nutritional interventions.

### Sleep

Sleep disorders are often a problem in ASDs, with prevalence rates ranging from 64 to 93% (62). The most common sleep problems are insomnia, increased bedtime resistance, sleep-disordered breathing, early morning awakenings, and daytime sleepiness (63). And it has been reported that ASD sleep disturbances are associated with SB and ASD severity (64–66). Here we present a report of a sleep disorder intervention that led to a reduction in SB and an improvement in SC.

The American Academy of Neurology published guidelines on sleep disturbances in ASD in 2020, which stated that melatonin, and cognitive behavioral therapy (CBT), alone or in combination, are highly effective in resistance to sleep, falling asleep, and staying asleep (67).

As noted above, there are many reports that provide evidence that sleep disturbances exacerbate ASD symptoms (68). On the other hand, not many reports examine whether sleep interventions changed SB and SC in ASDs. However, reports suggest that persistent melatonin improves self-aggressive internalizing behaviors such as withdrawal, anxiety, and depression (69), and interesting reports have emerged from telephone-based sleep consultant interventions for parents with children with coexisting ASD and ADHD, which have shown improvement in sleep problems and improvements in, SB, and SC (70).

### Interrelationships among the three pillars of “exercise,” “nutrition,” and “sleep”

From the above, it seems as if “exercise,” “nutrition,” and “sleep” each have an independent influence on ASD. However, I will briefly introduce the connection between the three elements, including the author’s thoughts.

Adequate nutrition is the foundation of exercise (physical activity), but the view may be broadened when “depression and stress” are intervened. Adequate nutrition and improvement of the intestinal environment have been shown to contribute to the improvement of depression and stress and may well be expected to increase social participation and physical activity. Increased physical activity has been suggested to regulate hunger for ASDs as well as for typically developing children (71), and it is expected to positively impact improving the eating habits of ASD. Moreover, this is not only true in the nutrition-to-exercise direction; depression and stress negatively affect exercise (physical activity), nutrition (appetite), and sleep. And given that exercise, nutrition, and sleep have each been shown to have a positive effect on depression and stress, and it can be assumed that each intervention interacts with the other. The effects of aerobic exercise have been reported to improve sleep efficiency, shorten sleep onset latency, and delay waking time after falling asleep (72, 73). Furthermore, there are reports that a combination of regular exercise and high protein intake (nutrition) contributes to good sleep quality (74). In addition, taking into account that gut microbiota and related metabolites have been suggested to be altered in ASDs sleep disorders, it is clear that the elements of “exercise,” “nutrition,” and “sleep” are inextricably linked (75).

## Conclusion

Until now, behavioral and psychological interventions have dominated most interventions for ASDs, but their impact has been limited. The exercise, nutrition, and sleep interventions presented in this review have been effective in improving social communication, and stereotypic behavior in ASDs in many cases and have the advantage of being less constrained by location and cost and can be started at any time. We hope that the interventions for ASDs will be more comprehensive and help people to have a richer social life.

## Author contributions

NY and KH: conception and design of the study, data collection, data analysis and interpretation, writing the manuscript, revising it critically for important intellectual content, final approval of the version to be submitted. All authors contributed to the article and approved the submitted version.

## Conflict of interest

The authors declare that the research was conducted in the absence of any commercial or financial relationships that could be construed as a potential conflict of interest.

## Publisher’s note

All claims expressed in this article are solely those of the authors and do not necessarily represent those of their affiliated organizations, or those of the publisher, the editors and the reviewers. Any product that may be evaluated in this article, or claim that may be made by its manufacturer, is not guaranteed or endorsed by the publisher.
